# Natural Deep Eutectic Solvents Enhance the Bioavailability and Antioxidant Activity of Oleanolic Acid in Self-Constructed Pickering High Internal Phase Emulsions

**DOI:** 10.3390/foods14203568

**Published:** 2025-10-20

**Authors:** Jie Yu, Chenjia Li, Qin Zhang, Benyang Li, Chaoxi Zeng

**Affiliations:** 1 Department of Food Science and Technology, College of Food Science and Technology, Hunan Agricultural University, No. 1 Nongda Road, Furong District, Changsha 410128, China; yuj123@stu.hunau.edu.cn (J.Y.); chenjiali@stu.hunau.edu.cn (C.L.); zhangq20001227@163.com (Q.Z.); 2 Xiangya School of Public Health, Central South University, 172 Tongzipo Road, Changsha 410013, China; 256901012@csu.edu.cn

**Keywords:** oleanolic acid, natural deep eutectic solvent, high internal phase emulsion, bioavailability, antioxidant

## Abstract

Oleanolic acid (OA)-stabilized water-in-oil Pickering high internal phase emulsions (HIPEs), using natural deep eutectic solvents (NADESs) as the internal phase (HIPE-NADES), were developed to enhance OA bioavailability. Three kinds of NADESs (proline: sorbitol (1:1), proline: glucose (1:1), and proline: glucose (5:3)) were selected, and HIPEs with pure water as the internal phase were used as the control group. In vitro digestion and Caco-2 models showed that HIPE-NADES significantly improved OA bioaccessibility via enhanced stability and solubility. Crucially, OA bioavailability reached 16.20–19.10%, markedly surpassing controls (*p* ≤ 0.05), indicating that NADESs’ hydrogen-bonding network facilitates intestinal uptake. In a t-BHP-induced Caco-2 oxidative stress model, OA-loaded HIPE-NADES significantly attenuated damage, reducing MDA and ROS while elevating GSH-Px, CAT, and SOD activities and GSH levels (*p* ≤ 0.05). NADESs themselves contributed substantially to antioxidant efficacy. HIPE-NADESs represent an effective platform for enhancing the bioavailability and bioactivity of hydrophobic phytochemicals like OA, enabling simpler and more stable delivery systems.

## 1. Introduction

Oleanolic acid (OA), a pentacyclic triterpenoid ubiquitously distributed in edible plants and medicinal herbs, exhibits broad-spectrum bioactivities, including antioxidant, hypoglycemic, antimicrobial, and anticancer properties [1], leading to its incorporation in dietary supplements and functional foods globally. Nevertheless, its therapeutic application is severely constrained by inherently low oral bioavailability, primarily attributable to poor aqueous solubility and limited membrane permeability, which substantially impede gastrointestinal absorption [2]. To address these limitations, two principal strategies have emerged: first, a structural modification to synthesize derivatives with enhanced pharmacokinetic profiles for drug development [3,4], and second, advanced encapsulation systems such as nanoparticles, liposomes, and solid dispersions designed to improve solubility and permeability [5]. Among encapsulation technologies, Pickering emulsions represent a rapidly advancing approach due to their straightforward fabrication, cost-effectiveness, and high encapsulation efficiency, with particle-stabilized interfaces conferring superior coalescence resistance compared to surfactant-based systems.

Numerous natural bioactives exhibiting low aqueous solubility, including β-carotene, curcumin, and anthocyanins, share delivery challenges analogous to OA. To enhance their bioavailability, researchers have engineered Pickering emulsions stabilized by synthetic or natural solid particles, such as silica, cellulose nanocrystals, proteins, chitosan, and alginate [6]. However, these exogenous stabilizers increase the formulation complexity and may not intrinsically enhance bioactive efficacy. This limitation can be addressed by exploiting the inherent interfacial activity of bioactives themselves to construct self-stabilized emulsion delivery systems. Preliminary work by our group confirms that low-solubility bioactives (e.g., ursolic acid (UA) and OA) can function as Pickering particles, forming stable water-in-oil (W/O) emulsions [7]. Such self-constructed systems not only significantly improve bioactive bioaccessibility but also confer structural resistance against gastric destabilization. Nevertheless, critical challenges persist regarding solubility limitations and cellular uptake barriers, which must be resolved to achieve optimal bioavailability and functional efficacy enhancement.

Natural deep eutectic solvents (NADESs) constitute an emerging class of green solvents comprising natural primary metabolites (including amino acids, sugars, and organic acids) that form liquids at ambient temperature through hydrogen-bond-mediated melting point depression [8]. These solvents demonstrate superior physicochemical properties unattainable by conventional organic solvents, coupled with exceptional solubilization capacity for low-water-solubility bioactives, thereby enhancing their functional potency in food matrices [9]. Oscar et al. [10] demonstrated that choline chloride-glycerol NADESs significantly enhanced both the extraction efficiency of borage bioactives and their in vitro bioavailability. Da Silva et al. [11] reported that NADESs (choline chloride:glycerol:citric acid at a 0.5:2:0.5 molar ratio) improved intestinal stability of phenolic compounds while increasing the bioavailability of blueberry-derived anthocyanins compared to conventional solvents. Sut et al. [12] documented that proline/malic acid/lactic acid/water NADESs (1:0.2:0.3:0.5) functioned as absorption promoters, elevating mouse plasma berberine concentrations and achieving 8-fold higher bioavailability versus aqueous suspensions. Complementary work by Saiswani et al. [13] established choline-based DESs as intestinal permeation enhancers that modulate tight junction proteins in Caco-2 monolayers, facilitating daptomycin transport. However, a mechanistic understanding of NADES-mediated bioactivity enhancement within emulsion delivery systems remains limited. Miao et al. [14] developed NADES-based (D-glucose:sucrose:water) Pickering emulsions, showing improved curcumin bioaccessibility during in vitro digestion. A comprehensive assessment of bioavailability enhancement in integrated delivery platforms warrants further investigation.

This study elucidates the influence of NADESs on OA bioactivity within emulsion delivery systems by incorporating NADESs into self-constructed OA-based HIPEs. We systematically investigate HIPE-NADESs as oral delivery vehicles to enhance the digestive stability of OA, improve intestinal absorption, and boost antioxidant efficacy. Three established NADES formulations were integrated into OA-stabilized HIPEs. Through an integrated approach combining in vitro simulated digestion, Caco-2 cell monolayer transport studies, and tert-butyl hydroperoxide (t-BHP)-induced oxidative stress modeling, we evaluated comparative delivery system performance. These investigations establish fundamental insights into HIPE-NADES systems for enhancing bioavailability and antioxidant activity of low-water-solubility bioactives while elucidating the underlying mechanistic pathways.

## 2. Materials and Methods

### 2.1. Materials

Oleanolic acid (OA, ≥98% purity) derived from loquat leaves was sourced from Shaanxi Jin Kang Tai Biotechnology Co., Ltd. (Weinan, China). Food-grade rapeseed oil was procured commercially. Hank’s Balanced Salt Solution (HBSS, pH 6.8) and porcine bile extract were obtained from Beijing Solarbio Technology Co., Ltd. (Beijing, China). Enzymes, including pepsin from porcine gastric mucosa (Cat# 77160, ≥400 units/mg), pancreatin (Cat# P7545, 8 × USP specifications), and lipase from porcine pancreas (Cat# L3126, ≥125 units/mg), were acquired from Sigma-Aldrich (St. Louis, MO, USA). Caco-2 cells (ATCC HTB-37) were supplied by Nanjing Ke Bai Biological Technology Co., Ltd. (Nanjing, China). Certified fetal bovine serum (FBS) originated from Shanghai Date Hill Biotechnology Co., Ltd. (Nanjing, China). Phosphate-buffered saline (PBS, pH 7.4), minimum essential medium (MEM), and penicillin–streptomycin solution were procured from Gibco Life Technologies (Grand Island, NY, USA). Trypsin-EDTA solution (0.25%) and Cell Counting Kit-8 (CCK-8) were purchased from Beijing Labgic Technology Co., Ltd. (Beijing, China).

### 2.2. Construction of the Digestive Medium

Simulated digestion samples comprised three formulations: ① Oleanolic acid (OA) dispersed in rapeseed oil (OA + oil, control group); ② water-in-oil (W/O) Pickering emulsions with an aqueous internal phase (HIPE-Water); and ③ NADES-based HIPEs with identical OA loading (HIPE-NADESs). All systems contained equivalent OA concentrations (1% *w*/*w*) and matching rapeseed oil content. The OA + oil blend was prepared by dissolving OA powder (1% *w*/*w*) in rapeseed oil under magnetic stirring (800 rpm) at 80 °C for 2 h. HIPE-Water and HIPE-NADESs were fabricated using a two-step homogenization protocol: first, the OA-saturated oil phase was mixed with the internal phase (Milli-Q water or pre-synthesized NADESs) at a 1:3 oil-to-internal phase ratio. Second, coarse emulsions were processed using an Ultra-Turrax T18 digital homogenizer (S18N-19G dispersing tool) (IKA Works, Staufen, Gemany) at 14,000 rpm for 6 min with intermittent ice-bath cooling to maintain 25 ± 2 °C.

### 2.3. In Vitro Digestion Analysis

#### 2.3.1. Oral Phase

According to the method of Liu et al. [15], with modifications, 2 g of emulsions (OA = 1 wt%, 0.5 g of rapeseed oil) were mixed with 7 mL NaCl (0.9% (*w*/*v*)), or 0.5 g of bulk rapeseed oil (OA = 1 wt%) was mixed with 8.5 mL NaCl (0.9% (*w*/*v*)). Briefly, 30 μL of 0.3 M CaCl_2_ and 970 μL of distilled water were added to a final volume of 10 mL of the digestion liquid. The samples were incubated in a water bath (37 °C, 200 rpm) for 10 min.

#### 2.3.2. Gastric Phase

Simulated gastric fluid (SGF) was prepared by mixing the oral phase digest with 8 mL NaCl, 5 μL CaCl_2_, and 1.0 M HCl to adjust the pH to 3.0. Pepsin (2000 U/mL) was dispersed in the SGF, and distilled water was added to a final volume of 20 mL of the digestion liquid. The samples were incubated in a water bath (37 °C, 250 rpm) for 2 h.

#### 2.3.3. Intestinal Phase

For stomach samples, 8 mL NaCl was added, and the pH was adjusted to 7.0 with NaOH (0.1 M~1 M). Then, 3.5 mL of bile salt solution (containing 187.5 mg bile extract, pH 7.0, PBS) and 40 μL of 0.3 M CaCl_2_ were added, and the pH of the samples was adjusted to 7.0. Pancreatin (100 U/mL) and lipase (1940 U/mL) were added to the samples. The samples were incubated in a water bath (37 °C, 250 rpm) for 2 h. The pH of the samples was maintained at 7.0 by the addition of 0.1 M NaOH, and the volume of 0.1 M NaOH consumed was recorded. The amount of total free fatty acids released from the simulated digestion was calculated by the following formula, according to the pH-stat method.

$$FFA\left(\%\right)=\frac{V_{NaOH}\times C_{NaOH}\times M_{Oil}}{W_{Oil}\times 2}\times 100\%$$

V_NaOH_ represents the volume of 0.1 M NaOH (mL) consumed by the titration of FFA; C_NaOH_ represents the concentration of the NaOH standard solution (0.1 M); M_Oil_ represents the average molecular weight of lipids (the relative molecular mass of rapeseed oil is 282.296); and W_Oil_ represents the total mass of oil initially present in the digestive system.

Following digestion, the sample was subjected to centrifugation at 5000 rpm for 10 min, after which the micellar phase was collected.

### 2.4. Measurement of Digestive Properties

#### 2.4.1. Particle Size Analysis Throughout the Digestion Process

Gastrointestinal stability of the three delivery systems was evaluated by monitoring the mean particle diameter (D_4,3_) and ζ-potential evolution using Malvern Mastersizer and Zetasizer instrumentation (Malvern PANalytical, Malvern City, UK). Digestates from gastric and intestinal phases were collected, diluted 1:100 in pH-matched aqueous solutions to prevent multiple scattering, and transferred to measurement cells for triplicate analysis. Microstructural evolution was tracked throughout digestion stages (pre-digestion, oral, gastric, and intestinal) via polarized light microscopy (Olympus BX53M (Olympus, Tokyo, Japan), 50× objective) using direct smear preparations on glass slides.

#### 2.4.2. Microstructural Analysis

Following incubation at the initial, oral, gastric, and small intestinal stages, a sample volume of 20 μL was carefully pipetted onto a microscope slide. The sample was then gently covered with a cover glass. Observation was subsequently carried out using an optical microscope equipped with a 50× oil immersion objective lens.

#### 2.4.3. Bioavailability

The micellar phase isolation protocol was adapted from Martin et al. [16], with modifications, wherein post-digestion samples underwent centrifugation (5000 rpm, 10 min, and 25 °C), yielding three distinct strata: an opaque sediment layer (bottom), a micelle-containing aqueous phase (middle), and undigested oil (top). The aqueous micellar fraction was syringe-aspirated (2 mL), combined with anhydrous ethanol (4 mL), sonicated (10 min), and membrane-filtered (0.22 μm PTFE). Oleanolic acid quantification employed HPLC (210 nm detection) using a YMC-Pack ODS column (250 × 4.6 mm, 30 °C) with a methanol mobile phase (1.0 mL/min flow rate; 20 μL injection volume). Triplicate analyses referenced a seven-point standard curve (20–1280 μg/mL OA) constructed from concentration–peak area correlations.

Bioaccessibility % = C_Micelle_/C_Digesta_ × 100%

C_Micelle_ represents the concentration of OA in micelles, and C_Digesta_ represents the concentration of OA in the entire sample.

### 2.5. Construction of a Caco-2 Cell Model

#### 2.5.1. Culture of Caco-2 Cells

Caco-2 cells were cultured in 4 mL of a complete medium, which consisted of 79% MEM, 20% FBS, and 1% penicillin–streptomycin solution. The cells were incubated at 37 °C in an atmosphere of 5% CO_2_ and 95% air. The culture medium was refreshed every 1–2 days. Once the cell confluence reached 80–90%, the cells were digested with trypsin for sub-culturing [17].

#### 2.5.2. Establishment of a Caco-2 Cell Monolayer

The Caco-2 cell model was established following Liang et al. [18], with modifications. Log-phase cells were washed twice with PBS, detached using 1 mL trypsin-EDTA (0.25%), and neutralized with 3 mL of minimum essential medium (MEM). After repeated pipetting to achieve single-cell dispersion, cells were counted via a hemocytometer (40× magnification) and resuspended in a complete growth medium at 1 × 10^5^ cells/mL. Cell suspensions were seeded onto 12-well or Transwell plates: a 0.5 mL apical chamber and a 1.5 mL basolateral chamber. The medium was replaced every 48 h (week 1) and daily thereafter until full differentiation at 21 days.

#### 2.5.3. Determination of the Transepithelial Electrical Resistance (TEER) of Caco-2 Cells

The transepithelial electrical resistance (TEER) of Caco-2 monolayers was monitored every 72 h throughout the 21-day differentiation period. Electrodes underwent 15 min of sterilization in 75% ethanol, air-drying, and HBSS rinsing. Prior to measurement, the culture medium was aspirated from apical (AP) and basolateral (BL) chambers, followed by gentle HBSS washing (×2). HBSS (0.5 mL AP, 1.5 mL BL) was replenished before inserting STX2 electrodes perpendicularly, namely, an apical probe positioned 1–2 mm above the monolayer without membrane contact and a fully immersed basolateral probe. Measurements commenced after 30 s stabilization (≤5% fluctuation), with monolayer integrity confirmed at TEER ≥500 Ω·cm^2^. The calculation formula for TEER is as follows:

TEER = (Ω − Ω_0_) × 1.12

In the formula, TEER represents the transmembrane resistance value of the Caco-2 cell membrane, in Ω·cm^2^; Ω is the transmembrane resistance of the cell membrane in the experimental group, that is, the transmembrane resistance with cells grown, in Ω; Ω_0_ is the resistance of the blank group, that is, the blank resistance without cells grown, in Ω; and 1.12 is the effective membrane area of the 12-well Transwell plate culture chamber, in cm^2^.

#### 2.5.4. Cytotoxicity Assay

Three experimental groups were established: a blank control (complete medium only), a vehicle control (cells without treatment), and an experimental group (cells + digested micelles). Cell viability following intestinal digest exposure was assessed via a CCK-8 assay [19]. Caco-2 cells (1 × 10^4^ cells/well, 100 μL) were seeded in 96-well plates and incubated (37 °C, 24 h). After the medium was aspirated, experimental wells received 100 μL MEM-diluted digestive fluid (25 μg/mL OA micelles), the vehicle control received a fresh medium, and the blank control received MEM. Following 24 h of exposure, wells were supplemented with 110 μL CCK-8 solution (1:10 dilution in MEM) and incubated (37 °C, 30 min), and absorbance was measured at 450 nm. The cell viability of each well was calculated according to the following formula:

Viability % = (OD_T_ − OD_K_)/(OD_C_ − OD_K_) × 100%

In the formula, ODₜ refers to the absorbance value of the experimental group; ODₖ refers to the absorbance value of the blank group; and OD_C_ refers to the absorbance value of the positive control group.

#### 2.5.5. Cellular Uptake

Based on the method of Liu et al. [15], with slight modifications, Caco-2 cells were seeded in 12-well plates at a density of 1 × 10^5^ cells per well. Following the removal of the MEM medium, the Caco-2 cell monolayer was washed thrice with HBSS (pH 6.8). The culture medium was refreshed every two days during the first week and daily during the second week, thereby obtaining the cell monolayer for TEER monitoring. Upon removal of the DMEM medium, the Caco-2 cell monolayer was washed three times with HBSS (pH 6.8). The cellular uptake of OA was investigated by adding 600 μL of an oil dispersion or emulsion digest containing an equivalent amount of OA to the cells, followed by incubation for 0.5, 1, 2, and 4 h. Following incubation, the cell monolayer was washed thrice with pre-cooled PBS to eliminate free samples. Subsequently, after harvesting the cells, the cell monolayer was detached by treating the cells with 600 μL of cell lysis buffer (PBS + 10% ethanol). Following sonication, the sample was centrifuged to obtain the supernatant, and the content of OA was quantified using HPLC. The cellular absorption rate of OA was calculated by the following equation:

Uptake% = M_cell_/M_micelle_ × 100%

In the formula, M_cell_ represents the amount of OA absorbed by the cells, and M_micelle_ represents the content of OA in the added micellar phase.

#### 2.5.6. Transcellular Transport

Caco-2 cells were seeded in 12-well plates at 1 × 10^5^ cells/well and cultured under standard conditions (37 °C, 5% CO_2_). The culture medium was replaced every 48 h during week 1 and daily from day 14 to day 21 until full differentiation [20,21]. Then, it was incubated at 37 °C and under 5% CO_2_. In the direction from the apical side to the basolateral side (AL-BL), 0.5 mL of digestive fluid was added, with the same OA concentration to the AL side, and 1.5 mL of blank HBSS was added to the BL side. At designated intervals (0.5, 1, 2, and 4 h), 100 μL aliquots were collected from basolateral chambers for HPLC quantification of the transported OA. The OA secreted by Caco-2 cells is defined as the ratio of the OA content at the receiving side to that of the initial OA.

Secretion% = M_Bas_/M_intial_ × 100%

Here, M_Bas_ represents the content of OA in the basal lateral solution, and M_initial_ is the content of OA before its transport.

#### 2.5.7. Calculation of the Bioavailability of OA

In accordance with the above experimental results of the transport of OA in the Caco-2 cell digestion system, the content of OA collected in the basolateral region after absorption and secretion by Caco-2 cells can be utilized to calculate the bioavailability of OA [22]. The calculation formula is as follows:

Bioavailability% = C_Basolateral_/C_Micelle_ × 100%

Here, C_Micelle_ represents the concentration of OA in the micellar phase, and C_Basolateral_ indicates the concentration of OA in the basolateral culture medium after treatment.

### 2.6. Antioxidant Activity of Cells

#### 2.6.1. Establishment of the Oxidative Stress Model of Caco-2 Cells

This method was modified with reference to Xu’s approach [23]. In each well of a 96-well plate, 100 μL of Caco-2 cell suspension (containing 1.5 × 10^4^ cells) was seeded and then incubated in a thermostatic incubator (37 °C, 5% CO_2_) for 24 h. Once the cells had adhered to the well surface, they were washed 2–3 times with pre-warmed PBS. Subsequently, t-BHP at different concentrations (100, 150, 200, 250, 300, and 350 μmol/L) was added to each well, and the plate was incubated in a thermostatic incubator (37 °C, 5% CO_2_) for 2 h to induce oxidative stress. Subsequently, cell viability was assessed using the CCK-8 assay (the same method described in Section 2.5.4). The optimal t-BHP concentration for inducing oxidative stress in Caco-2 cells was then identified.

#### 2.6.2. Measurement of MDA, SOD, GSH-Px, CAT, and GSH

Caco-2 cells (5 × 10^5^ cells/mL in 1 mL suspension) were seeded in 60 mm culture dishes and incubated (37 °C, 5% CO_2_, 24 h). Five experimental groups were established: a blank control (cells + MEM); an oxidative stress control (cells + 200 μM t-BHP; 3–5); and treatment groups (cells + t-BHP + 0.2% OA mixed micelles from OA + oil, HIPE-Water, or HIPE-NADESs). After 2 h of co-incubation, cells were PBS-washed, trypsinized (0.25%, 1 mL), and centrifuged (100× *g*, 5 min). Cell pellets were lysed in 1 mL ice-cold RIPA buffer (30 min, 4 °C), followed by centrifugation (14,000× *g*, 15 min, 4 °C). Supernatants were collected for biomarker analysis. OA mixed micelles were prepared by the in vitro digestion of lipid delivery systems and diluted to 0.2% in MEM based on cytotoxicity assays. MDA, SOD, GSH-Px, and CAT levels were quantified according to the manufacturer’s protocols (Cayman Chemical kits).

#### 2.6.3. Measurement of Reactive Oxygen Species (ROS)

Intracellular ROS levels were quantified via a H_2_DCF-DA assay [24] with Caco-2 cells seeded in black 96-well plates (2 × 10^4^ cells/mL, 100 μL/well) and incubated (37 °C, 5% CO_2_, 24 h). Experimental groups mirrored previous oxidative stress protocols: a blank control (MEM only), an oxidative control (200 μM t-BHP), and three treatment groups (200 μM t-BHP + 0.2% OA mixed micelles from OA + oil, HIPE-Water, or HIPE-NADESs). Micelles were prepared by digesting the respective delivery systems and were diluted to 0.2% in MEM based on the cytotoxicity data. Following 2 h of co-incubation, cells underwent PBS washing, 10 μM H_2_DCF-DA loading (30 min, 37 °C), and fluorescence measurement (λ_ex_ = 506 nm/λ_em_ = 526 nm) using a microplate reader, with the data normalized to control fluorescence intensity.

### 2.7. Statistical Analysis

All measurements were conducted in triplicate, and the experimental results were reported as the mean ± standard deviation. The data were analyzed by one-way analysis of variance (ANOVA) using SPSS27 Statistics. If the ANOVA structure indicated significant treatment effects, Duncan’s multiple range test was used to determine significant differences among treatments. Graphs were made using Origin2024 or GraphPad Prism9.5.1.

## 3. Results and Discussion

Building upon our group’s prior demonstration of OA-stabilized Pickering HIPEs exhibiting exceptional stability, this terpenoid-based emulsion system shows significant potential for addressing dissolution, delivery, and bioavailability challenges of poorly soluble terpenoids [7]. The concentration of OA we chose was 1% of the weight of the oil phase. A concentration of 1% is sufficient to enable its dual role as a Pickering stabilizer at the interface and as an active functional ingredient. Given the documented role of NADESs in enhancing dissolution and absorption of low-solubility phytochemicals, we incorporated three food-grade NADES formulations, namely, Pro:Sor (1:1), Pro: Glu (1:1), and Pro:Glu (5:3), which were selected for their established functionality in food applications [25]. Therefore, we systematically investigated NADES integration within OA-stabilized HIPEs to elucidate their impact on oral delivery performance.

### 3.1. Bioaccessibility of OA in Different Delivery Systems

Bioaccessibility constitutes a critical pharmacokinetic parameter for evaluating bioactive utilization from plant sources, directly correlating with lipid digestion efficiency. Using an in vitro digestion model, we assessed OA bioaccessibility and associated free fatty acid (FFA) release profiles across three delivery systems: unemulsified Oil + OA, HIPE-Water, and HIPE-NADESs.

**Figure 1:**
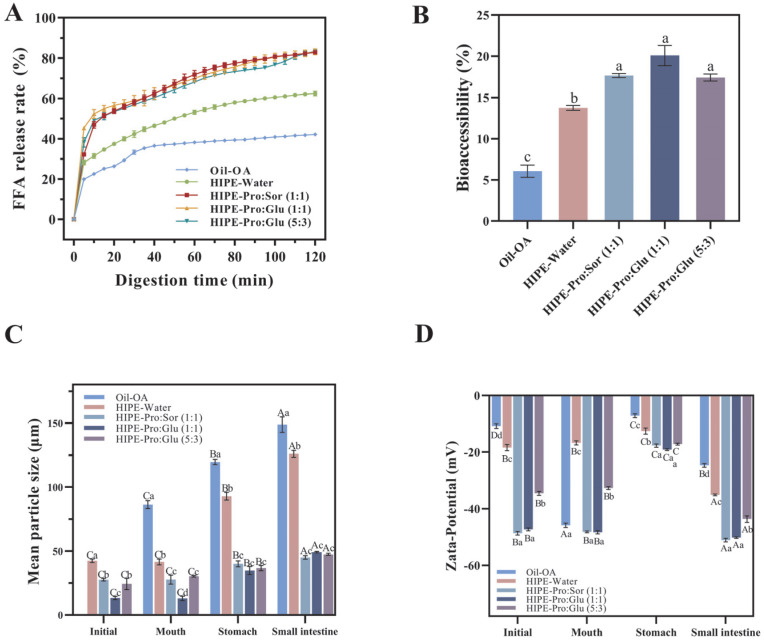
Free fatty acid (FFA) release profiles of three different delivery systems (Oil + OA (Oleanolic acid), HIPE-Water, and HIPE-NADESs) during simulated intestinal digestion (**A**). Bioaccessibility of OA derived from three different delivery systems (Oil + OA, HIPE-Water, and HIPE-NADESs) through in vitro simulated GI (Gastrointestinal) digestion (**B**), mean particle size (**C**), and Zeta-potential (**D**) of three different delivery systems (Oil + OA, HIPE-Water, and HIPE-NADESs) at each stage of digestion. (Different lowercase letters in indicate a significant difference of Bioaccessibility (**B**), mean particle siz (**C**), Zeta-potential (**D**) at *p* ≤ 0.05).

As demonstrated in Figure 1A, OA bioaccessibility followed the hierarchy of HIPE-NADESs (17.43–20.09%) > HIPE-Water (13.75%) > oil (6.07%). The HIPE-Water system exhibited 2.27-fold greater bioaccessibility versus unemulsified oil, confirming HIPE’s structural advantages for hydrophobic bioactive delivery. In the oil system, limited interfacial area restricted lipase–droplet interactions, consequently impeding OA transfer into micelles, a phenomenon corroborated by suppressed FFA release. The HIPE-NADES system significantly outperformed HIPE-Water (*p* ≤ 0.05), attributable to NADES-enhanced micellar incorporation via stronger polarity and hydrogen-bonding networks [9]. Critically, no significant differences emerged among NADES variants [Pro:Glu (1:1), Pro:Sor (1:1), and Pro:Glu (5:3)], consistent with their comparable solvent polarities.

Figure 1B illustrates FFA release kinetics during lipid digestion across delivery systems. All systems exhibited comparable release profiles: rapid initial FFA generation, followed by a gradual increase over time. Final FFA percentages followed the hierarchy of HIPE-NADESs (82.83–83.37%) > HIPE-Water (62.48%) > oil (42.23%), with oil showing the slowest kinetics and lowest yield. As lipid digestion is dependent on the interfacial process [26], HIPE architectures enhance the oil–lipase contact area, accelerating FFA release [27]. HIPE-NADES systems demonstrated significantly higher FFA release rates versus HIPE-Water (*p* ≤ 0.05), indicating NADES-mediated enhancement. This effect is mechanistically attributed to the superior gastric stability of HIPE-NADESs (Figure 1B), which minimizes droplet aggregation and preserves the interfacial area. Consequently, intestinal-phase lipolysis efficiency increases, facilitating OA solubilization [28]. This finding aligns with Miao et al. [14], where NADES-based HIPEs exhibited exceptional digestive stability and doubled micellar curcumin recovery versus unemulsified oil. Collectively, NADES integration optimizes HIPEs as efficient OA delivery vehicles through enhanced interfacial preservation and digestion kinetics.

The correlation between fatty acid release profiles and OA bioaccessibility across systems demonstrates direct dependency. During intestinal digestion, triglyceride hydrolysis generates di-/monoglycerides and, ultimately, FFAs, which form mixed micelles with bile salts. Emulsion systems enhance OA micellar solubilization within these hydrophobic domains, thereby increasing bioaccessibility [29]. In NADES-containing systems, accelerated triglyceride hydrolysis elevates FFA release rates. These FFAs function as endogenous surfactants that augment the micellar solubilization capacity for OA [30]. Wei and Huang [31] documented 14.6% higher bioaccessibility in Pickering emulsions (73.2%) versus organogels (63.9%), attributable to increased FFA-rich micelle generation. Crucially, a reduced emulsion droplet diameter promotes more rapid and complete lipid digestion, optimizing mixed micelle formation and, consequently, hydrophobic bioactive bioavailability [32].

To elucidate mechanistic differences in OA bioaccessibility, we characterized digesta particle size and ζ-potential evolution across delivery systems during simulated digestion (Figure 1). HIPE-NADES systems maintained significantly smaller particle sizes (*p* ≤ 0.05) throughout digestion stages compared to HIPE-Water and Oil-OA systems (Figure 1C), correlating with observed free fatty acid release kinetics. This size differential mediates interfacial lipase accessibility, directly influencing hydrolysis efficiency [33]. All systems exhibited a significant increase in gastric-phase particle size (*p* ≤ 0.05), attributable to pH-mediated charge modulation. Upon transition from oral (neutral pH) to gastric (acidic) phases, proton influx reduces the hydroxyl ion concentration, diminishing the droplet surface charge (Figure 1D). The consequent attenuation of electrostatic repulsion promotes droplet aggregation. Crucially, HIPE-NADES systems maintained |ζ-potential| >30 mV versus ≤30 mV in HIPE-Water, indicating sufficient electrostatic stabilization to mitigate coalescence [34]. This charge preservation underlies the enhanced structural integrity of NADES-containing systems during gastric stress.

The HIPE-NADES system exhibited significantly reduced particle size versus HIPE-Water (*p* ≤ 0.05), demonstrating superior resistance to gastric destabilization. This size preservation confers critical functional advantages: during intestinal transit, the maintained colloidal dimensions provide a greater interfacial area for bile salt adsorption and lipase accessibility, accelerating micelle formation and lipid digestion kinetics. Mechanistically, this stability enhancement is attributed to a higher density of OA solid particles at the oil–NADES interface compared to oil–water systems. The resultant stronger electrostatic repulsion between droplets effectively restricts aggregation phenomena. This finding aligns with Chang et al. [35], who reported analogous instability in rhamnolipid-stabilized emulsions under acidic conditions (pH ≤ 3), where impaired emulsifier adsorption capacity led to interfacial failure and flocculation.

### 3.2. Bioavailability of OA in Different Delivery Systems

#### 3.2.1. Assessment of Cytotoxicity and Establishment of the Caco-2 Cell Model

**Figure 2:**
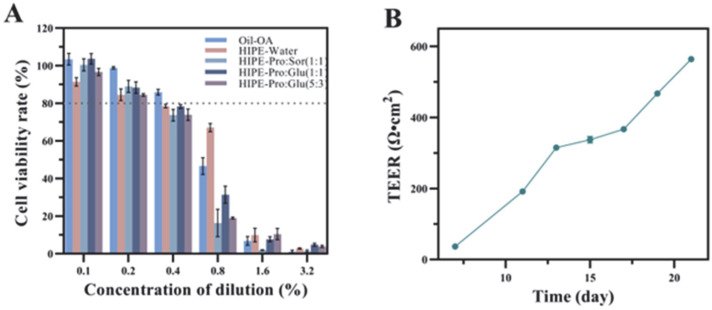
Cell viability of Caco-2 cells exposed to micelles at different concentrations was assessed by CCK8(The dotted line indicates a cell viability of 80%) (**A**). The resistance value of the Caco-2 cell monolayer after incubation for 21 days, (**B**).

The Caco-2 cell monolayer closely recapitulates the functional characteristics of human small intestinal epithelium, establishing its utility as a validated model for investigating bioactive absorption mechanisms [22]. Prior to transport studies, we assessed micellar phase biocompatibility through CCK-8 cytotoxicity screening (Figure 2A). Experimental concentrations were optimized at 0.2% dilution, maintaining >80% cell viability across all test conditions. Crucially, the three NADES formulations exhibited excellent safety profiles, consistent with literature reports, showing no statistically significant cytotoxicity differences compared to NADES-free HIPE controls (*p* > 0.05). For absorption analysis, cell monolayers were cultured for 21 days until transepithelial electrical resistance values above 500 Ω·cm^2^ were achieved (Figure 2B), confirming intact barrier function. These validated monolayers were subsequently employed to evaluate OA uptake and transport kinetics following the in vitro digestion of each delivery system.

#### 3.2.2. Effects of Different Delivery Systems on the Bioavailability of OA

**Figure 3:**
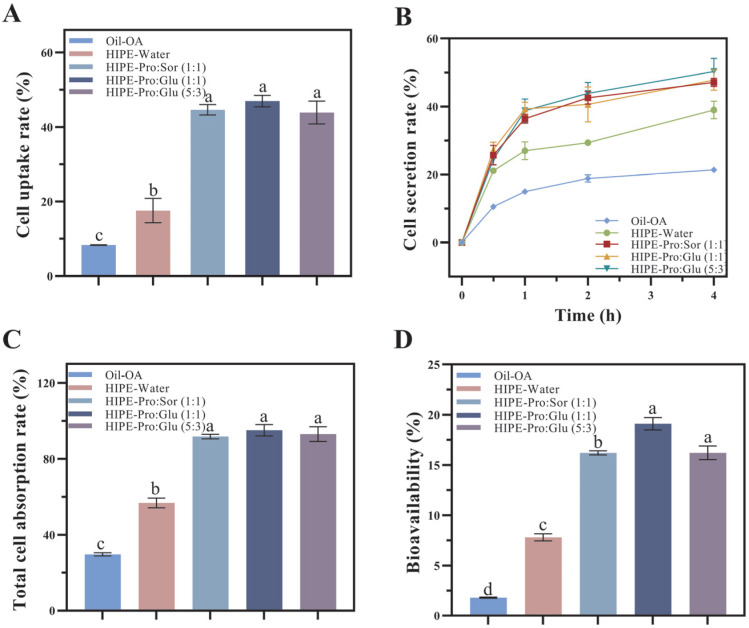
The effects of different delivery systems on cell uptake (**A**), secretion (**B**), total uptake (**C**), and bioavailability (**D**) were evaluated using the Caco-2 monolayer model. OA(Oleanolic acid), HIPE (High internal phase emulsion), Pro (Proline), Sor (Sorbitol), Glu (Glucose). (Different lowercase letters in indicate a significant difference of cell uptake (**A**), total uptake (**C**), bioavailability (**D**) at *p* ≤ 0.05).

Following gastrointestinal transit, cellular uptake profiles were assessed using micellar phases derived from the three delivery systems. OA uptake from micelles was quantified by culturing Caco-2 cells. Total cellular absorption (Figure 3C) was calculated as the sum of cellular uptake (Figure 3A) and basolateral secretion (Figure 3B). Delivery system properties significantly influenced OA absorption and transport. Minimal OA absorption occurred from the oil system (29.70% total absorption). HIPE-based carriers markedly enhanced OA uptake, with HIPE-Water achieving 56.75% absorption. Crucially, the internal phase composition substantially affected absorption outcomes. Compared to HIPE-Water, NADES-containing systems significantly enhanced both cellular uptake and basolateral secretion (*p* ≤ 0.05), resulting in total absorption rates of 91.73–95.08%.

As shown in Figure 3A, the cellular uptake of OA from the HIPE-Water system (17.59%) was significantly higher than from the oil system (8.34%; *p* ≤ 0.05), demonstrating that the HIPE structure enhances cellular absorption efficiency. The HIPE-NADES systems exhibited substantially greater uptake (43.88% to 46.94%), representing a 2.49- to 2.67-fold increase relative to HIPE-Water. This significant enhancement confirms that NADES incorporation potentiates the cellular uptake efficiency of OA delivered via HIPEs.

Mechanistically, hydrophilic NADES components (glucose, sorbitol, and proline) migrate toward micellar surfaces during digestion. Both glucose and sorbitol contain multiple hydroxyl groups that collectively enhance the hydrophilicity of micellar outer layers. Intestinal absorption efficiency is fundamentally governed by molecular proximity to the brush border membrane. The hydroxyl-enriched surfaces of HIPE-NADES micelles facilitate diffusion through the unstirred water layer, enabling a closer approach to absorptive surfaces. Consequently, this hydrophilic modification significantly enhances cellular internalization of OA-loaded micelles. These findings align with Lu et al. [36], who demonstrated that migration of hydrophilic groups (specifically glycerol moieties) to micellar surfaces enhances hydrophilicity, promoting cellular penetration and increasing bioactive uptake rates.

The secretion of OA across the Caco-2 monolayer into the basolateral compartment was quantified through transport experiments (Figure 3B). All OA formulations exhibited time-dependent secretion profiles. After 4 h of incubation, significant differences (*p* ≤ 0.05) in OA secretion were observed across delivery systems, attributable to micellar property variations generated during in vitro digestion. These properties directly modulate OA transport efficiency across intestinal epithelia.

The oil system showed minimal OA secretion (21.36%), while HIPE-Water demonstrated 83.05% higher secretion (39.01%; *p* ≤ 0.05). This enhancement aligns with HIPEs’ capacity to promote OA dissolution in simulated intestinal fluid (SIF) and amorphous incorporation into mixed micelles, facilitating cellular transport [22]. Crucially, HIPE-NADES systems achieved 47.81–50.32% secretion—representing a 22.56–28.99% increase over HIPE-Water (*p* ≤ 0.05)—confirming that NADES significantly potentiates HIPEs’ secretory enhancement. Mechanistically, NADES-imparted micellar hydrophilicity creates a hydrophilic “stealth” layer that reduces recognition by efflux transporters (e.g., P-glycoprotein). As P-glycoprotein preferentially extrudes hydrophobic substrates, this surface modification decreases efflux-mediated clearance, prolonging intracellular retention of OA-loaded micelles and, consequently, increasing basolateral secretion.

**Figure 4:**
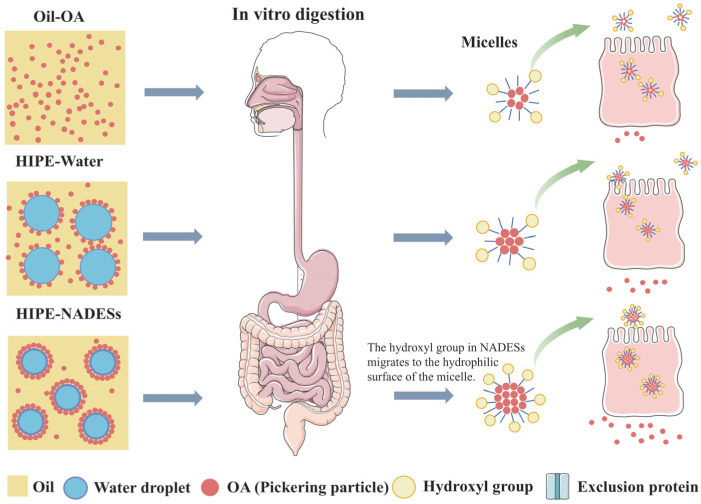
Schematic illustration describing the possible procedures of the digestion and small intestine epithelial cellular uptake of OA.

Figure 4 illustrates the effects of three oleanolic acid (OA) delivery systems on OA bioaccessibility and bioavailability, evaluated through integrated in vitro digestion simulations and Caco-2 monolayer transport assays. Bioavailability (F), defined as the fraction of OA reaching systemic circulation, is calculated as F = F_A_ × F_B_, where F_A_ represents the cellular absorption fraction (Figure 3C) and F_B_ denotes micellar bioaccessibility (Figure 1B). The bioavailability hierarchy demonstrated statistically significant differences (*p* ≤ 0.05): HIPE-NADESs (16.20–19.10%) > HIPE-Water (7.80%) > oil (1.80%). HIPE-based systems substantially outperformed the oil control, with NADES-containing variants exhibiting superior bioavailability relative to aqueous HIPEs. The constrained bioavailability in HIPE-Water originated from concurrent limitations in both absorption efficiency (F_A_ = 56.75%) and bioaccessibility (F_B_ = 13.75%). NADES enhances bioavailability through dual synergistic mechanisms: first, by modulating lipid digestion kinetics to accelerate lipolysis and mixed micelle formation, and second, via hydroxyl-rich components (glucose/sorbitol) engineering hydrophilic micellar surfaces that facilitate cellular penetration through unstirred water layer traversal while competitively inhibiting P-glycoprotein recognition via hydrophobic domain obstruction. This surface modification reduces efflux transporter affinity, prolonging intracellular micellar retention and enhancing transcellular transport. Consequently, HIPE architectures represent optimal delivery vehicles for hydrophobic bioactives like OA in functional foods, with NADES integration providing paramount bioavailability enhancement through these physicochemical and biological interactions.

### 3.3. Antioxidant Activity

**Figure 5:**
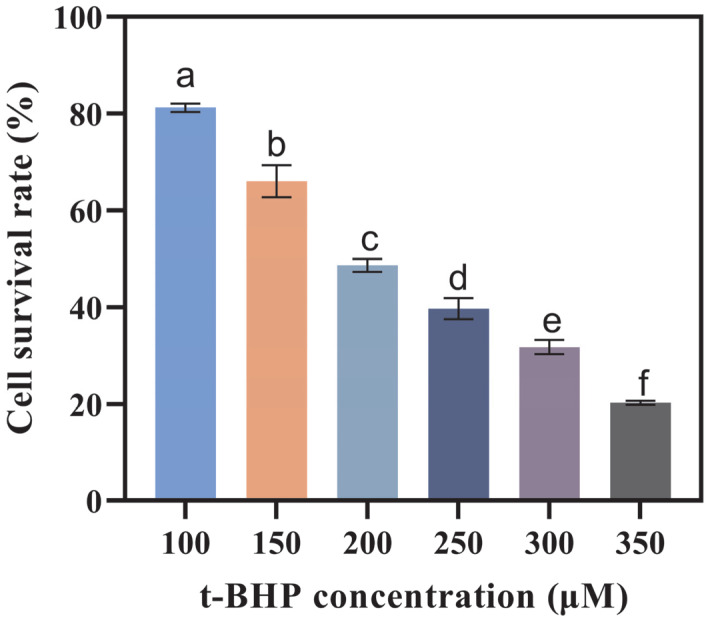
Cell viability of Caco-2 cells after being induced by different concentrations of t-BHP (tert-Butyl hydroperoxide) for 2 h. (Different letters indicate significant difference at *p* ≤ 0.05).

**Figure 6:**
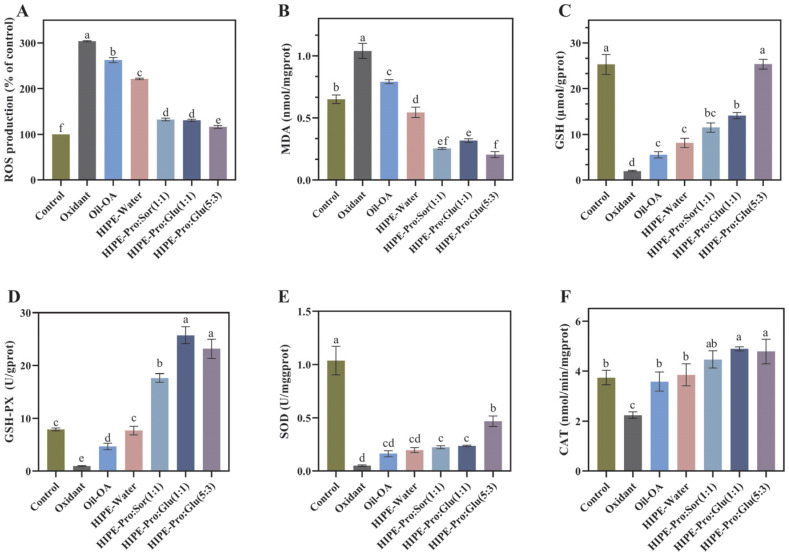
Effects of OA (Oleanolic acid) mixed micelles formed by three delivery systems on the levels of intracellular ROS (Reactive Oxygen Species) (**A**), MDA (Malondialdehyde) (**B**), GSH (glutathione) (**C**), GSH-PX (Glutathione peroxidase) (**D**), SOD (Superoxide dismutase) (**E**), and CAT(Catalase) (**F**) in Caco-2 cells induced by t-BHP. (Different lowercase letters in indicate a significant difference of ROS (**A**), MDA (**B**), GSH (**C**), GSH-PX (**D**), SOD (**E**), CAT (**F**) at *p* ≤ 0.05).

To assess the health benefits of OA-containing mixed micelles formed by three delivery systems following in vitro digestion, an oxidative stress model was developed in Caco-2 cells using t-BHP for induction. T-BHP serves as a crucial factor contributing to oxidative damage. It has a longer half-life compared to other reactive oxygen species and can readily be converted into hydroxyl radicals, which are among the most destructive free radicals [37]. As the concentration of t-BHP increased, the viability of Caco-2 cells decreased significantly (Figure 5). When the concentration of t-BHP reached 200 μmol/L, the survival rate of Caco-2 cells was 48.62%. As depicted in Figure 6, in comparison to the control group, the content of malondialdehyde (MDA) in the model group exhibited a 59.89% increase. Meanwhile, the enzymatic activities of superoxide dismutase (SOD), glutathione peroxidase (GSH-PX), and catalase (CAT) decreased by 95.09%, 87.78%, and 40.09%, respectively (*p* ≤ 0.05). Moreover, the intracellular reactive oxygen species (ROS) level increased significantly, suggesting the successful establishment of the model.

In order to assess the impact of OA mixed micelles formed by three delivery systems following in vitro digestion on the oxidative stress of Caco-2 cells, we measured the oxidative stress markers in Caco-2 cells. SOD, CAT, and GPX are three major enzymatic antioxidant defense enzymes. GSH is part of the non-enzymatic antioxidant defense system. To a great extent, they determine the antioxidant defense ability of the human body. The synergy between the enzymatic and non-enzymatic antioxidant defense systems serves to prevent oxidative stress, thereby averting damage to cellular structures and functional impairments. MDA [38] is a degradation product of lipid peroxidation induced by oxidative damage. It can be used as an indicator of the level of lipid peroxidation.

As shown in Figure 6, in comparison to the oxidant group, the MDA and ROS levels in the three HIPE-NADES delivery systems exhibited a notable decline. Specifically, the ROS levels nearly recovered to those of the control group, whereas the MDA levels were markedly lower than those of the control group. In comparison to the oxidant group, the activities of GSH-Px, CAT, GSH, and SOD in the three HIPE-NADES delivery systems were all markedly enhanced. Among these, the most prominent one was the HIPE-Pro: Glu (5:3) system. When compared to the oxidant group, following pretreatment with the OA mixed micellar phase formed by this system, the MDA and ROS contents in cells decreased by 80.28% and 61.96%, respectively (*p* ≤ 0.05). Additionally, the activities of superoxide dismutase (SOD), glutathione peroxidase (GSH-Px), catalase (CAT), and glutathione (GSH) increased by 8-fold, 18-fold, 1-fold, and 11-fold, respectively (*p* ≤ 0.05). Interestingly, when compared to the control group, the activities of GSH-Px and CAT in the three HIPE-NADES delivery systems also exhibited notable increases. This suggests that NADES can significantly enhance the antioxidant capacity of the system, as it not only counteracts the negative effects of t-BHP. In terms of enhancing the activities of GSH and SOD, HIPE-Pro: Glu (5:3) demonstrated a greater antioxidant advantage than the other two NADESs. To more expediently elucidate the antioxidant-influencing factors of the HIPE-NADES delivery system, the experiment incorporated a group with the addition of pure oil and a HIPE-Water group. In comparison to the oxidant group, both the MDA and ROS contents in the cells of the Oil-OA group and the HIPE-Water group exhibited a notable decrease (*p* ≤ 0.05). Meanwhile, the activities of GSH-Px, CAT, and GSH displayed a significant increase (*p* ≤ 0.05). The results indicated that both OA itself and the HIPE system made certain contributions to the antioxidant capacity of the overall system. The antioxidant property of OA itself is not difficult to comprehend, and there have been numerous extensive studies on its antioxidant mechanism.

The antioxidant capacity of the HIPE emulsion system may be because the special structure of the emulsion allows the antioxidant capacity of OA to be more comprehensively exhibited. Rudra Pangeni and colleagues reported that a nanoemulsion loaded with resveratrol was more efficient in decreasing the MDA level within cells and elevating the activities of SOD, GSH-PX, and CAT compared to free resveratrol. The HIPE delivery system incorporated with NADESs exhibits a remarkable antioxidant capacity. This phenomenon may be attributed to the components of NADESs, namely, proline, sorbitol, and glucose. These components are capable of exerting antioxidant effects independently and synergistically interact with OA to enhance the overall antioxidant activity [39]. Furthermore, the presence of NADESs enhances the hydrophilicity of OA-containing mixed micelles. This property aids OA in surmounting its inherent hydrophobicity and poor permeability, thereby facilitating the full manifestation of OA’s antioxidant capabilities. The HIPE-NADES system exhibited superior antioxidant activity. Specifically, the CAT activity reached the highest level, which indicated an enhanced ability to scavenge hydrogen peroxide. This type of intervention may effectively mitigate oxidative stress and confer protective effects against oxidative damage to organs. The elevation in GSH-PX activity further evidences a more robust antioxidant defense system. This enhancement is particularly evident in alleviating lipid peroxidation. The elevated SOD activity and the decreased MDA level suggest efficient scavenging of superoxide radicals and a reduction in cell membrane damage.

## 4. Conclusions

This study successfully developed an OA-stabilized HIPE system by replacing the aqueous phase with NADESs, termed HIPE-NADESs. Through integrated in vitro digestion simulations and Caco-2 cell models, we demonstrated that NADES incorporation significantly enhances OA bioavailability and antioxidant efficacy. Crucially, HIPE-NADESs exhibited superior digestive stability versus OA-in-oil systems, evidenced by smaller droplet sizes (*p* ≤ 0.05), elevated FFA release rates, and 46–52% higher bioaccessibility relative to HIPE-Water. The Caco-2 transport assays revealed 2.1- to 2.4-fold greater bioavailability (16.20–19.10%) for HIPE-NADESs compared to HIPE-Water (7.80%), attributable to hydrogen-bond-mediated OA solubilization in micelles and the enhancement of micellar hydrophilicity by hydroxyl-rich NADES components. These modified micelles demonstrated 1.8-fold increased transcellular transport efficiency (*p* ≤ 0.05) due to improved unstirred water layer penetration and reduced efflux transporter recognition. Collectively, HIPE-NADESs represent a breakthrough delivery platform for hydrophobic bioactives, combining exceptional digestive stability with enhanced bioavailability through dual mechanisms of solubility augmentation and membrane interaction optimization, thereby establishing a versatile strategy for functional food and nutraceutical applications requiring simplified, yet high-performance, carrier systems. The NADES emulsion system developed in this study provides a promising platform technology for improving the delivery of hydrophobic active ingredients, such as oleanolic acid. Its application can be extended to the fields of pharmaceuticals, functional cosmetics, and functional food. The natural, economical, and safe raw materials used in this system, along with the simple production process, give it excellent potential for industrialization. Subsequent research will focus on formula optimization and in vitro/in vivo evaluations to facilitate the commercialization process.

## Data Availability

The original contributions presented in this study are included in the article. Further inquiries can be directed to the corresponding author.
